# Continuity with caveats in anesthesia: state and response entropy of the EEG

**DOI:** 10.1007/s10877-024-01130-9

**Published:** 2024-04-03

**Authors:** Max Ebensperger, Matthias Kreuzer, Stephan Kratzer, Gerhard Schneider, Stefan Schwerin

**Affiliations:** 1 Department of Anesthesiology and Intensive Care, School of Medicine and Health, Technical University of Munich, Ismaningerstr. 22, 81675 Munich, Germany; 2 Abteilung für Anästhesiologie, Intensiv- und Schmerzmedizin, Hessing Stiftung, Hessingstraße 17, 86199 Augsburg, Germany

**Keywords:** Anesthesia, Electroencephalogram, Burst suppression, State entropy, Response entropy

## Abstract

The growing use of neuromonitoring in general anesthesia provides detailed insights into the effects of anesthetics on the brain. Our study focuses on the processed EEG indices State Entropy (SE), Response Entropy (RE), and Burst Suppression Ratio (BSR) of the GE Entropy^TM^ Module, which serve as surrogate measures for estimating the level of anesthesia. While retrospectively analyzing SE and RE index values from patient records, we encountered a technical anomaly with a conspicuous distribution of index values. In this single-center, retrospective study, we analyzed processed intraoperative electroencephalographic (EEG) data from 15,608 patients who underwent general anesthesia. We employed various data visualization techniques, including histograms and heat maps, and fitted custom non-Gaussian curves. Individual patients’ anesthetic periods were evaluated in detail. To compare distributions, we utilized the Kolmogorov–Smirnov test and Kullback–Leibler divergence. The analysis also included the influence of the BSR on the distribution of SE and RE values. We identified distinct *pillar *indices for both SE and RE, i.e., index values with a higher probability of occurrence than others. These* pillar *index values were not age-dependent and followed a non-equidistant distribution pattern. This phenomenon occurs independently of the BSR distribution. SE and RE index values do not adhere to a continuous distribution, instead displaying prominent *pillar * indices with a consistent pattern of occurrence across all age groups. The specific features of the underlying algorithm responsible for this pattern remain elusive.

**Supplementary Information:**

The online version contains supplementary material available at 10.1007/s10877-024-01130-9.

## Introduction

### Background

To monitor the hypnotic component of anesthesia, the electroencephalograph (EEG) is a non-invasive measurement that directly monitors anesthetic-induced changes in the target organ, the brain [1, 2]. Commercial monitoring systems use a reduced EEG montage to record from the patient’s forehead. The systems translate the raw EEG into an index, which provides information regarding the patient’s anesthetic level, i.e., the hypnotic component of anesthesia [3–6]. Most of these monitoring systems use algorithms to detect changes in the EEG spectrum by tracking the transition from a low-amplitude, high-frequency EEG present in the awake state to a high-amplitude, low-frequency EEG during anesthetic-induced unconsciousness [1]. The monitoring systems translate these changes into dimensionless indices that inversely correlate with the hypnotic component of anesthesia. These indices are mostly proprietary, and we only have partial insight into how these indices are derived [3–7]. Results from reverse-engineering one of the most commonly used systems, the Bispectral Index^TM^ (BIS), indicate that even the partially provided information may be misleading [8]. Other monitoring systems, like the GE Entropy^TM^ Module with the State Entropy (SE) and the Response Entropy (RE), appear more transparent, as parts of the algorithms were explained [4, 9]. The SE and RE seem to be calculated from the spectral entropy [4, 10], which is the Shannon Entropy [11] applied to the power spectrum of the EEG. Another article explains the steps for calculating the Burst Suppression Ratio (BSR) [9]. The BSR information is straightforward to interpret, it indicates the percentage of suppressed EEG duration within a defined period. The *depth-of-anesthesia* indices are more complex because the output of the respective mathematical algorithm is matched on a scale ranging from 0 to 100 for RE or – as in the case of SE – to 91 [4]. Nonetheless, the exact mechanism by which the algorithm executes this matching process remains obscure.

### Objectives

In our retrospective data analysis, our primary aim was to investigate the presence of age-dependent distribution patterns in SE and RE index values. While examining the large dataset comprising SE, RE, and BSR data, we discovered an unanticipated clustering of particular *pillar* index values, a finding which we elaborate on further in this article. These findings challenge the prevailing claim that SE and RE index values originate from a continuous matching of spectral entropy values onto a *depth-of-anesthesia* scale [4].

## Methods

### Electronic patient records

For this retrospective, single-center study, we performed an analysis of processed intraoperative EEG data from a cohort of 15,608 patients who underwent general anesthesia. Our hospital’s electronic records contained index values of SE, RE, and BSR from the GE Entropy^TM^ module. Additionally, these records included demographic information such as patient age, sex (male or female), Body Mass Index, ASA Physical Status Classification and whether inhalation anesthesia with volatile anesthetic gases or total intravenous anesthesia (TIVA) was used for anesthesia maintenance. Furthermore, the records featured multiple perioperative procedural timestamps and time periods, outlining the total perioperative time. This includes: start of anesthesia, anesthesiological case clearance, surgical incision, and end of anesthesia, in accordance with the German Perioperative Procedural Time Glossary [12]. We included all patients aged 18 and older in our analysis. The trend data (RE, SE, and BSR) were recorded at 10-second intervals. All analyses and plots were computed using MATLAB R2023a [13].

### Definition of time intervals for analysis

For certain aspects of our analysis, we selected specific time intervals to isolate periods during which patients can be expected to reach a stable state of general anesthesia. To define this interval, we adjusted two of the procedural timestamps to better align with our research objectives. Specifically, we used the timestamp *surgical incision* as a reference point and subtracted 5 min to delineate the beginning of our target period from anesthesia induction with more dynamic pharmacological interventions and concomitant EEG changes. To exclude patients who were awakening from anesthesia, we subtracted 10 min from the *end of anesthesia* timestamp. This approximated differentiation allowed us to evaluate the indices for (i) the entire procedural period (including states of patient wakefulness and awakening in the operating room) and (ii) the surgical core period with likely stable anesthesia-induced unconsciousness.

### Analysis of SE and RE index values

As previously mentioned, both SE and RE index values are derived from spectral entropy analysis. The frequency ranges used for calculating these indices are 0.8–32 Hz for SE and 0.8–47 Hz for RE, as specified in Viertio-Oja et al.’s work [4]. Notably, RE encompasses information from higher-frequency components, capturing a greater extent of recorded muscle activity. In this study, we present the results for both SE and RE separately, as well as their respective difference for each index value pair, $\Delta$ (RE–SE). This difference contains valuable information, as a $\Delta$ (RE–SE) > 10 might be associated with impending arousal [4, 14]. Both RE and SE are initially computed as spectral entropy values. However, in order to ease interpretation by clinicians, these values are converted into whole integer indices, ranging from 0 to 91 (SE) or 100 (RE). This transformation is purportedly accomplished via application of a monotonic spline function, which is chosen to ensure that the resulting curve is perfectly smooth, devoid of any abrupt changes or irregularities, while still preserving the essential information. It’s important to note that this transformation does not evenly space the values from the original scale. This deliberate choice is made to optimize data resolution within the SE original range of 0.5 to 1.0 [4].

### Statistical analysis

For parts of our analyses, we focused on the surgical core period only, because including the values from awake or awakening patients during the entire procedural period with high SE and RE index values would have constituted a biasing factor for our distribution fits. Throughout this study, we explicitly indicate whether the data presented are derived from the entire procedural or the surgical period. We visualize our findings with histograms, heat maps, and trends. In order to calculate probabilities of index value distributions, the data is initially portioned into discrete intervals known as bins. Subsequently, the data points within each of these bins are enumerated. This is followed by a normalization step, where the counts within each bin are divided by the total count of data points. This ensures that the sum of probabilities equals 1. For the indices’ distribution during the surgical period, we created an expected, continuous probability distribution by fitting a custom, interpolated, non-Gaussian curve to the histogram of observed probabilities and then subtracted the *pillar* index values above the fit. For this purpose, we applied the MATLAB linear interpolation function *interp* to estimate values for the vacant positions of excluded *pillar* index values. After the fitting, the custom curve was transferred back to the original histogram. This adjustment was made for SE values (2 to 76; manually chosen for visual fit optimization) to provide a more accurate representation of the *pillar* index values. The expected probability distribution was then renormalized. Observed and expected distributions of probabilities were compared using Kullback–Leibler divergence as an information-based measure of disparity (MATLAB function *klDiv*), [15] and the Kolomogorov–Smirnov test as a nonparametric goodness-of-fit test for distributions, using a significance threshold of *p* < 0.05; (MATLAB function *kstest2*), [16]. We further fitted mono-exponential functions [17]. Due to the non-parametric nature of distributions, all values are presented as medians with 25th (Q1) and 75th (Q3) percentiles or as percentages (%) in case of categorical variables.

### Bias

The process of visually recognizing *pillar* index values could potentially introduce unintended biases. Nevertheless, we have taken deliberate steps to mitigate this by explicitly specifying and illustrating the SE and RE index values that we have categorized as *pillar* values and we list them here for the surgical period (3, 5, 6, 7, 9, 11, 12, 14, 15, 16, 18, 20, 22, 24, 25, 26, 27, 28, 29, 30, 31, 33, 35, 37, 41, 45, 51, 59, 66, 72, 77). This transparency extends to our use of expected distribution fits, which includes non-Gaussian modified fits. These measures are aimed at providing a clearer demarcation between the observed distribution and the expected distribution.

### Missing data

We aimed to clean our large dataset by removing any fully or partially incomplete or invalid index values pertaining to SE, RE, and BSR. This was achieved by implementing exclusion criteria, as demonstrated and detailed in Fig. S1. Additionally, all *NaN* values, which in MATLAB represent non-real or complex numbers, were automatically disregarded in all arithmetic functions as well as in all statistical analyses.

## Results

### Patient characteristics

The median age of the included patients was 59 [43–72] years. 54% were male and 46% were female. The median surgery duration was 120 [79–179] minutes. Due to the high percentage of incomplete data sets, we excluded any surgery exceeding a duration of 8 hours (273 out of 15,550 patients). A flowchart showing all exclusion criteria is presented in Fig. S1. The corresponding histograms with surgery durations and age distributions are presented as supplemental Fig. S2.

### Distribution of SE and RE index values

#### Overall distribution of SE and RE

**Fig. 1:**
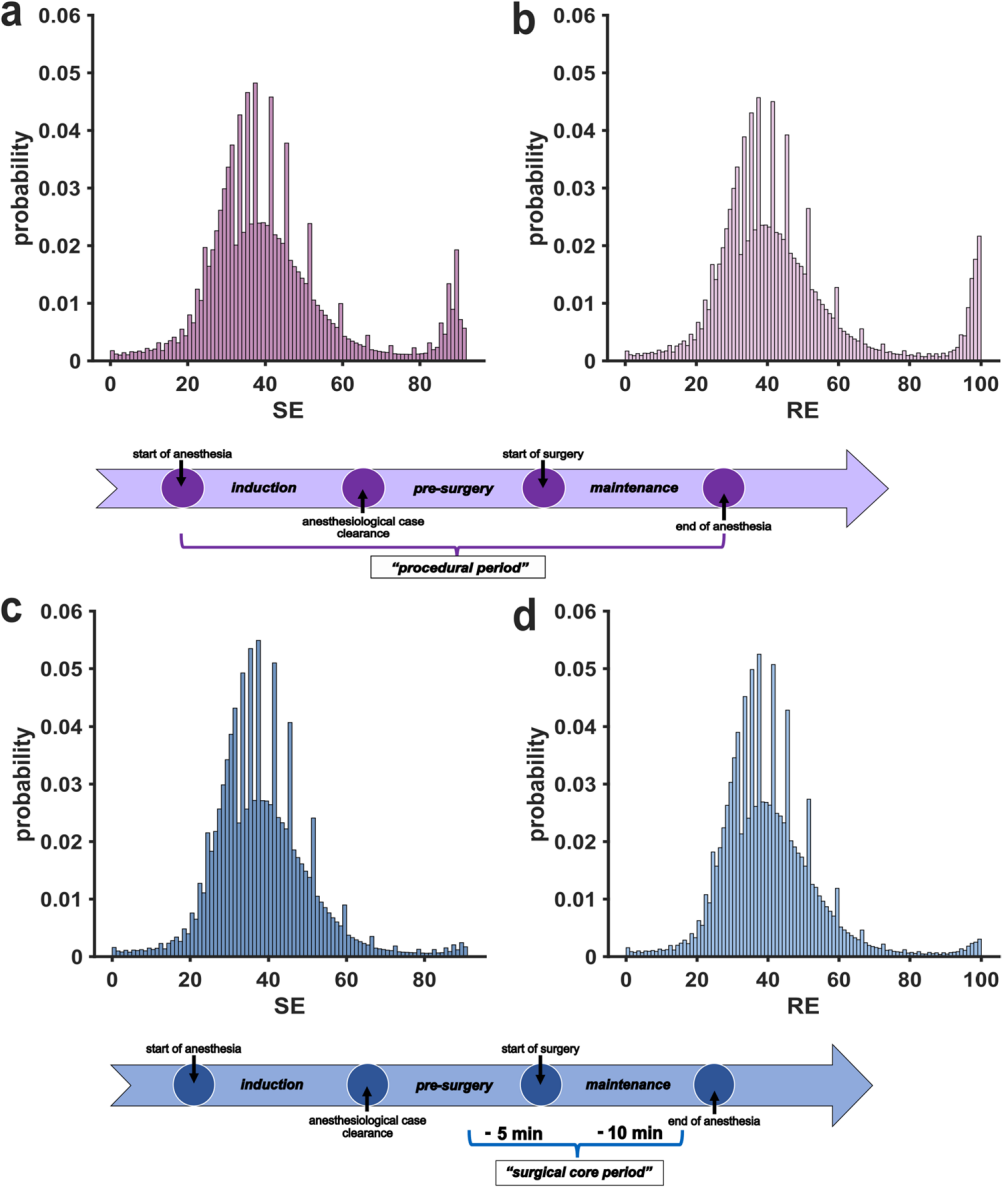
**Histograms of state entropy (SE) and response entropy (RE) probability distributions for all age groups**. Multiple index values show a distinctively higher probability compared to adjacent values and contrast the expected continuous index value distribution. Below the histogram, respective timelines indicate the relevant time points and periods. **a** Histogram of probabilities for SE index values during the procedural period. **b** Histogram of probabilities for RE index values during the procedural period. **c** Histogram of probabilities for SE index values during the surgical period. **d** Histogram of probabilities for RE index values during the surgical period

The first step comprised the visualization of the available index data. Figure 1 contains the histograms that show a bimodal probability distribution for the SE and RE data over the entire procedural period and all age groups combined with index accumulation in the range to be expected under general anesthesia (<60) and in the awake range (>90) (a, b). When only considering the surgical period, the number of high index values (>80) strongly decreases (c, d), as expected. Within these distributions, distinct values for both SE and RE with a sharply increased probability in comparison to directly adjacent values can be visually identified. These peak values, which we term *pillar* indices, were not distributed isometrically. This observation suggests that the algorithm outputs are not equally matched onto the 0–91 (SE) or 0–100 (RE) scale.

#### Distribution of SE and RE index values stratified by age

**Fig. 2:**
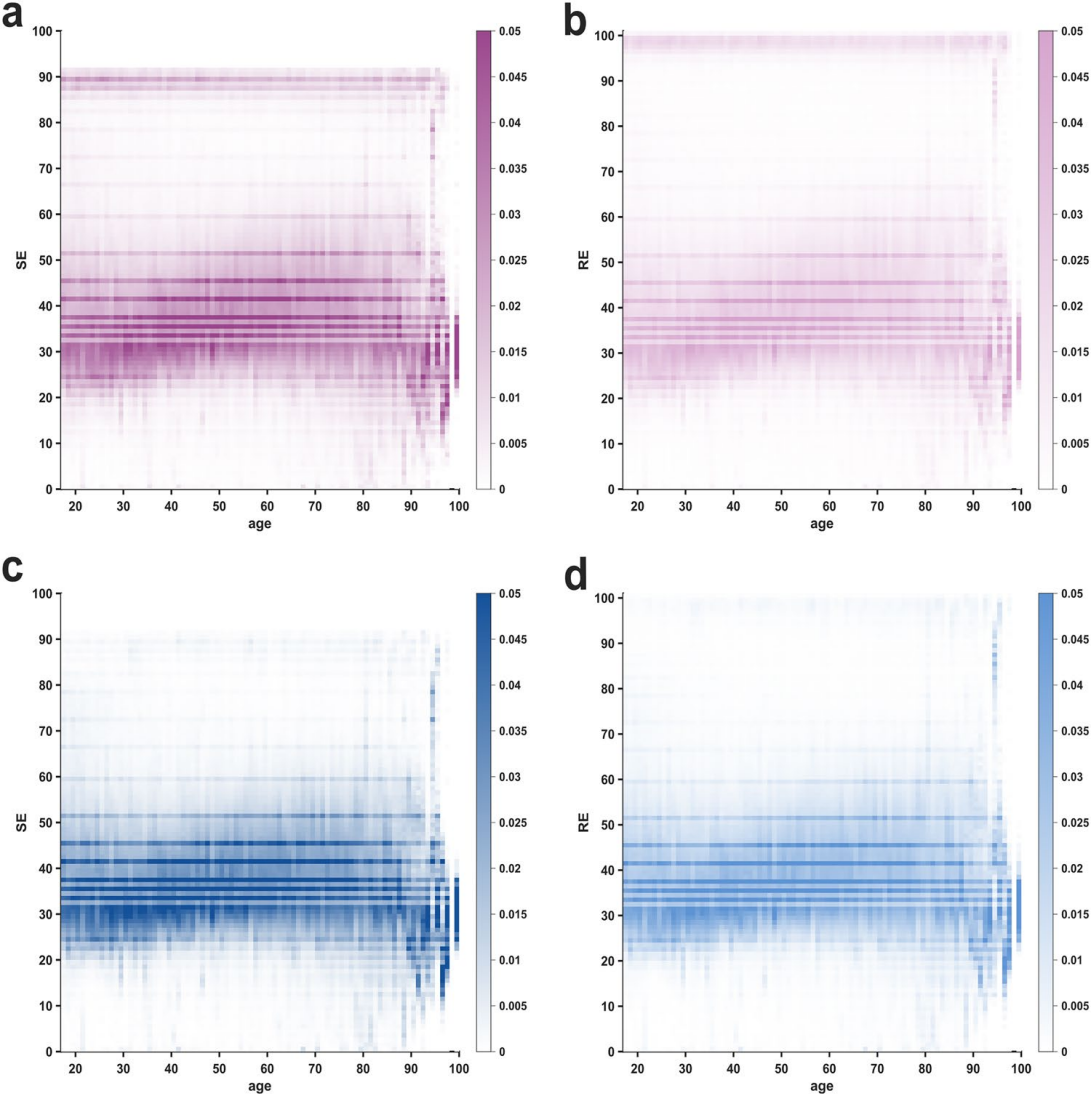
**Heat maps of state entropy (SE) and response entropy (RE) probability distributions as a function of age**. The horizontal, darkened lines indicate a distinctively higher probability occurrence for the respective SE and RE index values as well as a high consistency over the entire age range. Greater data dispersion in the age groups over 90 years is attributed to the limited sample size of patients above 90 years of age. **a** Heat map of SE index values during the procedural period. **b** Heat map of RE index values during the procedural period. **c** Heat map of SE index values during the surgical period. **d** Heat map of RE index values during the surgical period

The heat maps in Fig. 2 show the index densities as a function of age for the entire procedural period (a, b) and only the surgical period (c, d). We observed perfectly horizontal, high-density lines in the heat maps, denoting a significant concentration of particular index values. These values are identical to the *pillar* indices observed in the histograms. The distinct lines show a consistently higher occurrence probability for SE and RE values across all age groups. Their occurrence therefore appears to be independent of patient age, i.e. to be of technical nature. The marked change in distribution patterns beyond the age of 90 years can be attributed to the very limited number of patients included in these age groups, which leads to stronger data dispersion or missing values altogether. As in the histograms, the horizontal lines follow the same, non-isometrical distribution pattern over the index range. Especially in older patients (c, d), we found higher probabilities for very low index values (<30) and high, *awake* values (>80). We also present the heat maps of the differences between RE and SE in supplemental Fig. S3. Notably, the $\Delta$ (RE–SE) does not display distinct *pillar* index values. Instead, the variation appears to be spread in a more continuous manner. This observation suggests that there is a perfect overlap in the *pillar* values between SE and RE.

#### Analyzing individual trends of SE and RE index values

**Fig. 3:**
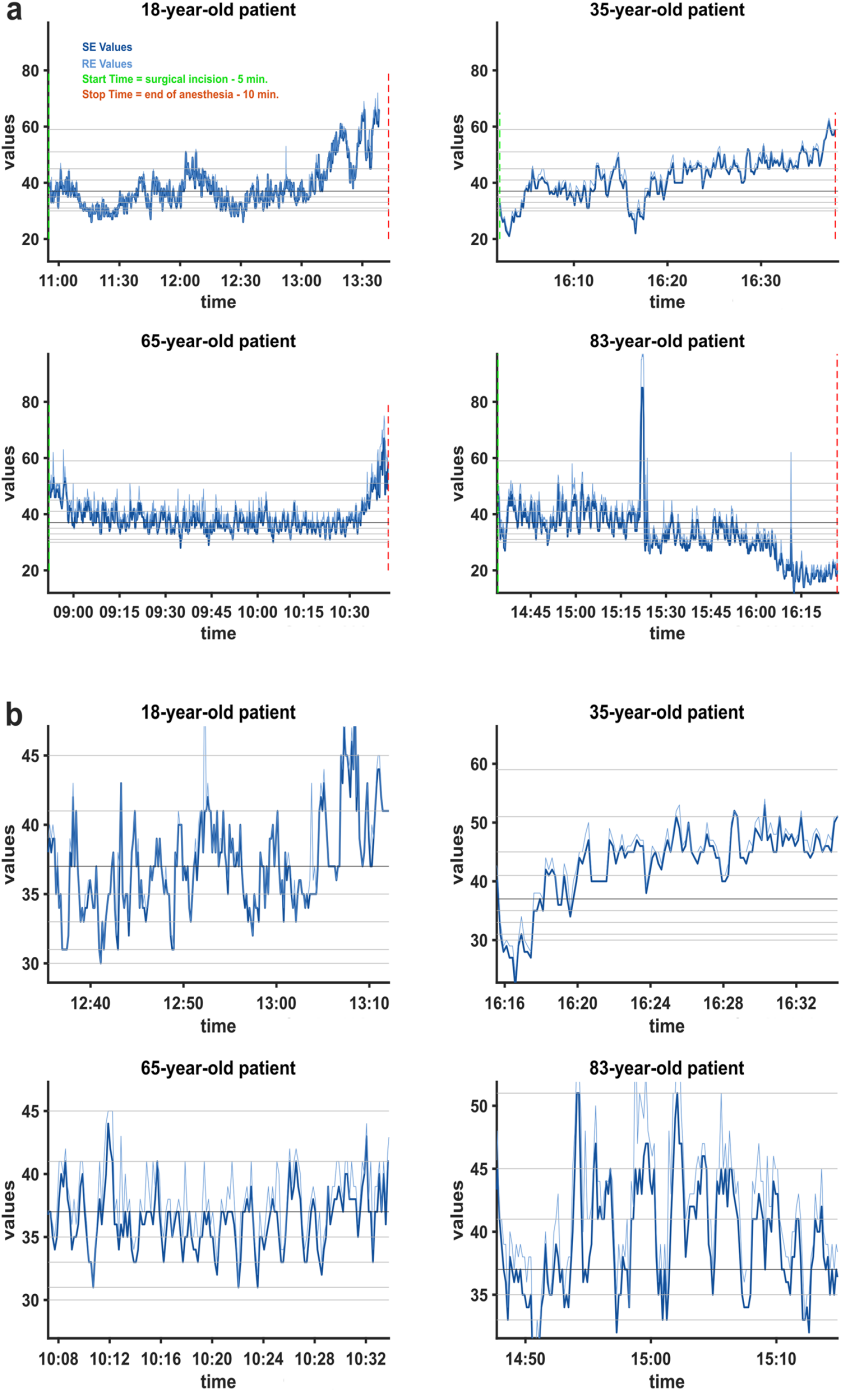
**Trend data of state entropy (SE) and response entropy (RE) values recorded during the surgical period from four exemplary patients of different age groups**. SE and RE data are presented in dark and light blue. Procedural timestamps are marked out vertically and *pillar* index values are indicated as grey horizontal lines. **a** Trend data for four patients throughout the entire surgical duration showed no discernible *pillar* indices and fell within the anticipated value range. **b** Sections with increased temporal resolution from the same four patients. Visible are plateauing values as well as peak values, which appear to constitute turning points and coincide with *pillar* index values

The histograms and heat maps revealed that *pillar* indices occur much more frequently compared to adjacent index values. To elucidate this phenomenon and to discern the underlying mechanisms, we further analyzed the SE and RE frequency distributions. How SE and RE occurrence behaves on an individual level is exemplified in Fig. 3. It shows the SE and RE trend data of different patients during their respective surgical interventions with two different time scales. The trends covering the entire surgical period show a continuous course of the indices, which seemingly fluctuate freely in the range recommended for anesthesia (40–60) and there is no indication of the described *pillar* index values, see Fig. 3a. However, upon examination of the trends with increased temporal resolution, we noticed that the indices appear to linger at certain values for a prolonged duration. Furthermore, the indices exhibit peak points with subsequent trend reversion, which appear to coincide with *pillar* index values and may account for their more frequent occurrence (see Fig. 3b).

### Time distribution across specific SE and RE index values

#### Maximum durations and consecutive runs for SE and RE index values

**Fig. 4:**
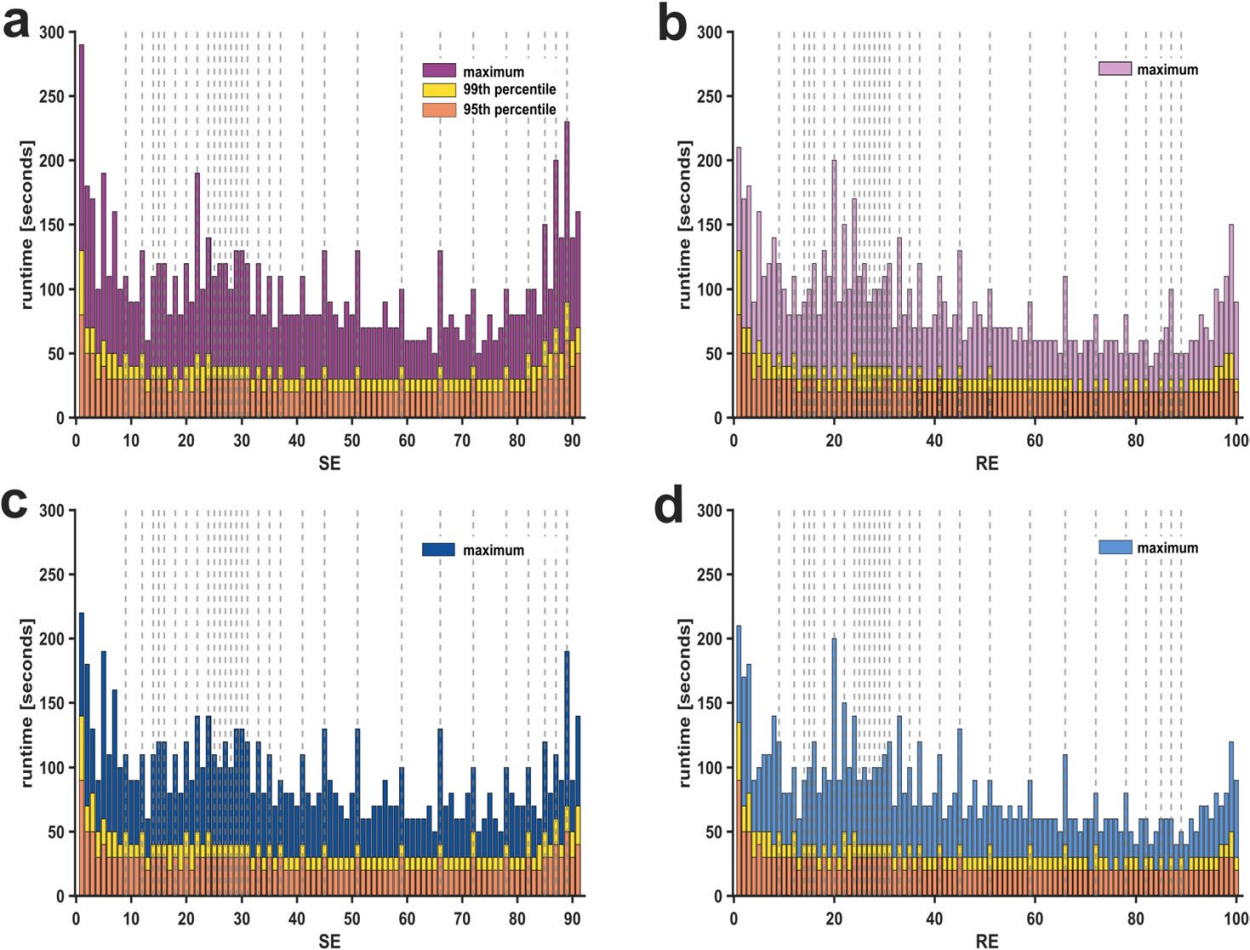
**Maximum consecutive runtime spent in state entropy (SE) and response entropy (RE) values for the entire procedural period and the surgical period**. The time spent in specific SE and RE index values indicated as consecutive runs with 10-second epochs per run. Maximum time spent, 99th, and 95th percentile are displayed separately. The previously identified *pillar* index values are highlighted with vertical grey dotted lines. **a** Consecutive runs of SE indices in the procedural period. **b** Consecutive runs of RE indices in the procedural period. **c** Consecutive runs of SE indices in the surgical period. **d** Consecutive runs of RE indices in the surgical period

We further evaluated the cumulative time spent in individual index values and the maximum duration, during which an index value did not change. The histograms in supplemental Fig. S4 show that the cumulative time spent in the previously identified *pillar* indices was, as one might expect, much higher. The maximum duration during which the index remained unchanged was also higher in the case of *pillar* index values. In Fig. 4, we display the maximum consecutive runs (10-second epochs per run) of individual SE and RE index values, both for the entire procedural and the surgical period, respectively. Because it may be argued that the maximum run duration was influenced by artifacts, we also included the 99th and 95th percentiles.

#### Consecutive occurrence of *pillar* indices and adjacent index values

Having established that *pillar* SE and RE index values occur with greater frequency and longer duration than other index values, we extended our analysis. We compared these *pillar* indices to directly adjacent index values and plotted the number of observations against consecutive occurrences. Both *pillar* index values and the respective adjacent index values are well described by negative exponential functions and show excellent fits (R^2 ^> 0.99). However, *pillar* index values demonstrate differences in both the rate of decay as well as the vertical shift. Across different segment lengths, *pillar* indices were over-represented compared to adjacent indices. Further details can be found in the supplemental Fig. S5 and Table S1, which contains the parameters of the exponential fits.

### Interpolation for SE index values during the surgical period

**Fig. 5:**
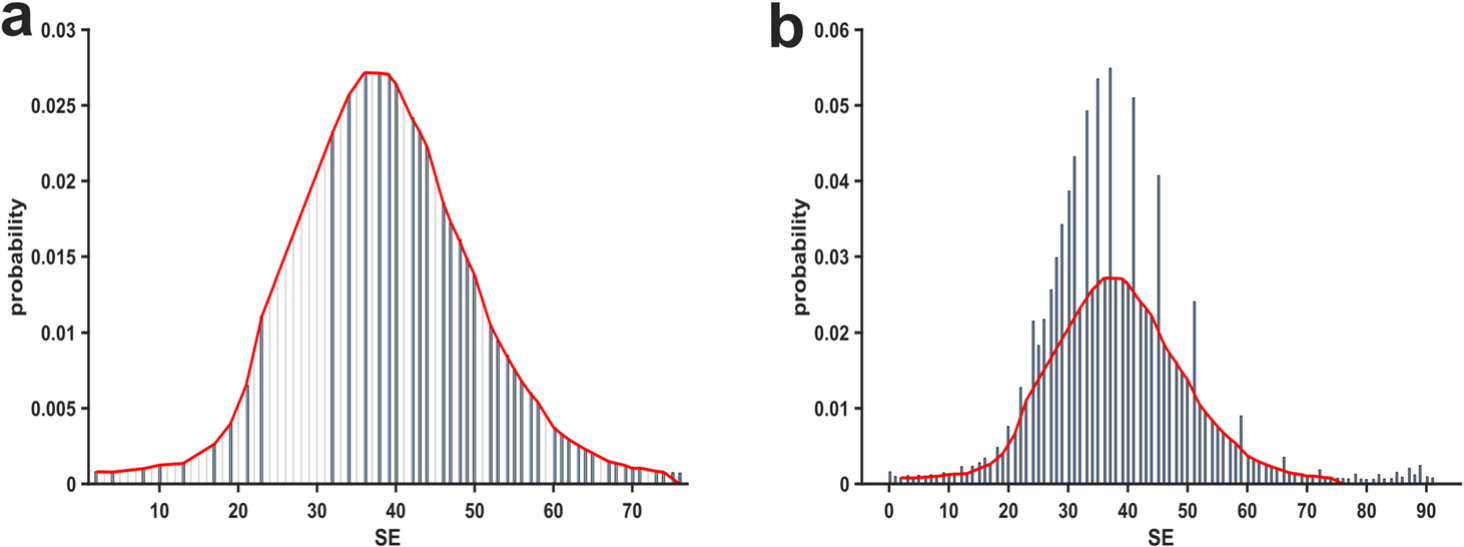
**Fit modeling and peak index identification of State Entropy (SE) index values.** **a** Histogram of the SE index values that were included to create a custom, non-gaussian fit, superimposed as a red line. **b** Histogram of SE index values including *pillar* index values and the modeled fit superimposed in red, which was used to quantify the excess probability of *pillar* index values

To evaluate the percentage of *pillar* index values above an expected distribution (presupposing a pattern of continuously increasing probabilities towards a peak value before continuously decreasing), we manually modeled a fit based on the (visually identified) non-peak values, as shown in Fig. 5a. The fit reveals a nearly symmetrical distribution around a maximum point of SE equal to 36. The fraction of excess probability above the fit as depicted in Fig. 5b was 25.46% for SE index values during the surgical period.

### Analyzing distributional discrepancies: Kullback–Leibler divergence and Kolmogorov–Smirnov tests

In order to evaluate the differences between observed and expected distributions, we first calculated the Kullback–Leibler divergence (KL), which was 0.048. To contextualize this metric more meaningfully, we also tested the interpolated values against a linear, *ideal* value distribution, resulting in a KL divergence of 0.465. Additionally, we compared the observed probability distribution against the same linear, ideal probability distribution, obtaining a KL divergence of 0.553. These results indicate that the interpolated probability distribution only deviates by approximately one-tenth from the observed distribution in comparison to linear values. Furthermore, we performed the Kolmogorov–Smirnov test twice. First, we used the *Ksstat* function to compare the measured probability distribution against a normal distribution (CDF), which produced a KS statistic (D) of 0.176 (*p* = 0.018). This significant difference between the two distributions suggests that the measured probabilities differ significantly from a normal distribution. Secondly, we used the *Kstest2* to compare the measured probabilities against our interpolated probabilities. In this analysis, the KS statistic (D) was found to be 0.095 (*p* = 0.879), indicating that the probability distribution of the observed data is consistent with the one of interpolated, *expected* probability distribution.

### Evaluation of burst suppression ratio (BSR)

**Fig. 6:**
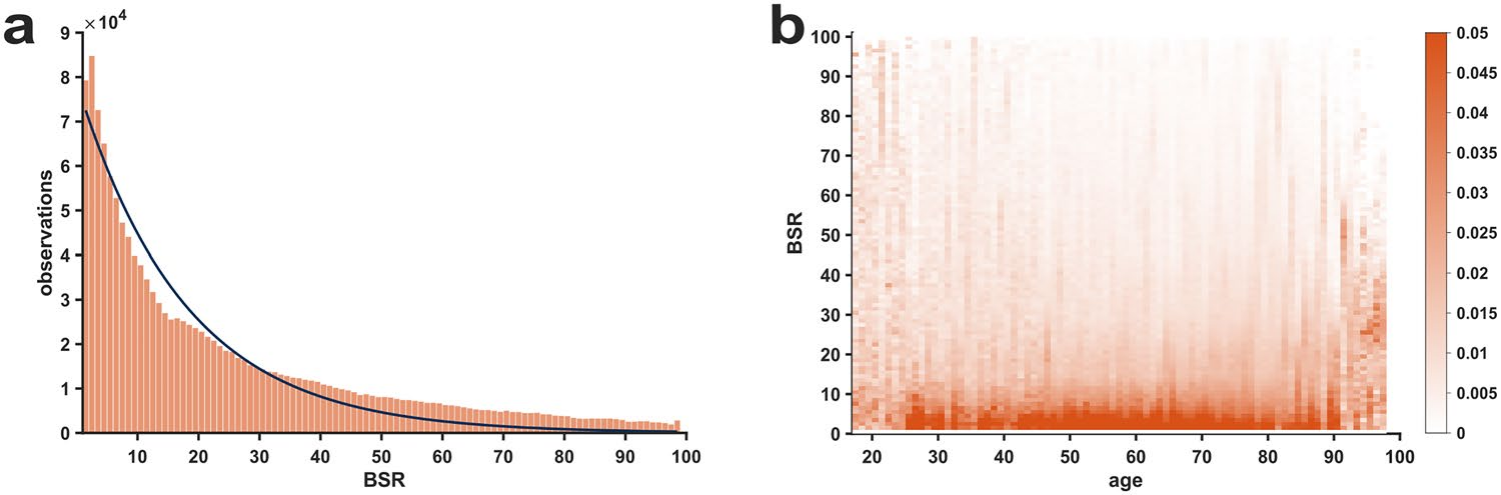
**Distribution of Burst Suppression Ratio (BSR) indices > 0 for the entire procedural period**. For clarity, BSR of 0 was omitted as it accounted for approximately three-fourths of all BSR values. **a** Histogram showing the count distribution of BSR values > 0. The decay appears continuous without discrete *pillar* indices and can be well described by an exponential decay function, which is superimposed in the figure. **b** Heat map of BSR > 0-probability distribution, stratified by age, that shows a continuous fading of probability with increasing BSR index values

When evaluating the BSR index with the same histogram and heat map approach as SE and RE for the entire procedural period, we found that the BSR did not show *pillar* index values, but a rather continuous decline in occurrence probability, which could be well described with an exponential decay function (R^2 ^=  0.948), as depicted in Fig. 6a. The heat map visualizing the probability distribution of BSR values across the age range showed a continuous behavior without horizontal lines, in contrast to RE and SE. The BSR heat map is presented in Fig. 6b. To prevent scaling distortions in the plots, only BSR values>0 are included. This is because the most frequently observed BSR value was zero, representing absence of any burst suppression as detected by the algorithm, and accounted for 74.24% of the whole BSR dataset. When plotting the BSR indices as a function of SE indices, the *pillar* indices are readily discernible as horizontal lines, while there are no vertical lines for BSR, further suggesting a continuous distribution of BSR values, see Fig. S6.

### Exploration of potential confounders

To identify potential confounders, we further investigated the distribution of SE/RE index values depending on Body Mass Index, ASA Physical Status Classification, surgery department, and the choice between the use of volatile anesthetic gases or total intravenous anesthesia (TIVA) for the surgical period. In our analysis, the *pillar* index values persisted for all conditions, and no confounding factors could be identified. The histograms showcasing the distribution of SE/RE values grouped according to volatile anesthetic gases versus the use of TIVA are presented in Fig. S7.

## Discussion

Every manufacturer of EEG-based monitoring systems emphasizes the primacy of processed index values for navigating the level of anesthesia. The utilization of these indices, like BIS and other systems, was discussed as a means to prevent intraoperative awareness [18], although some publications show contradicting results [19, 20]. Other publications have demonstrated that these indices show a (mostly unknown) temporal lag [21, 22], which may depend on artifact content and in part on the electromyographic component [23, 24], or that they overlook or misinterpret burst suppression [25–27]. While the algorithms generating these indices are proprietary, other freely accessible parameters have demonstrated effective – and in some cases even superior – performance in monitoring the anesthetic level [28, 29]. Therefore, relying on an index whose precise design is not fully disclosed may not be the most advisable approach to establish trust both in clinical practice and research settings, particularly in light of these limitations. There are publications that have uncovered substantial components of the BIS algorithm via reverse engineering, showing its reliance on the low-gamma waveband and contradicting earlier beliefs about its dependence on a bispectral index [8, 30]. Additionally, there have been instances highlighting contradictory information provided by simultaneous BSR and SE outputs [31].

Our results point towards discrepancies between the prevailing explanation of how the SE/RE indices are calculated and what we can observe clinically. As stated in the manuscript describing the algorithm [4], the spectral entropy is matched onto the 0–91 (SE) or 0–100 (RE) scale per spline interpolation. Subsequently, the spline function is characterized as smooth without “kinks”. This leads to the assumption that the index distribution was also intended to be smooth and not contain *pillar* index values with overly high occurrence probability, which we found in our dataset and describe in this study. We could also observe that in the RE and SE range of approximately 30 to 90, the *pillar* index values were further apart from each other than in the lower index range. For the higher range with values between 50 and 90, the presented spline function in the description paper was also steepest [4].

While the clinical implications of our findings have yet to be explored in further detail, they could indeed impact the use of the indices for research purposes. Different monitoring systems were shown to provide discordant recommendations based on identical EEG recordings with patterns of emergence from anesthesia [32]. Much like other clinical parameters that require monitoring during general anesthesia — such as electrocardiography — the interpretation and classification guiding clinical decisions should not be contingent upon the specific monitoring device employed. Further studies have described discrepancies between processed EEG monitoring devices, for example, simultaneously recorded BIS and SE index values during general anesthesia with sevoflurane [33]. The underlying uncertainties and deviations from the expected behavior of processed EEG indices might be particularly problematic when studies compare or aim for the identical target value range with different monitoring systems [34]. Comparability is further impeded by the observation that processed EEG indices, specifically SE/RE and BIS demonstrate an extensive inter-individual variability with regard to defined clinical endpoints as well as anesthetic-specific differences [35]. Another study describes that SE and RE values are not well matched by two different BIS target corridors [36], which might be partially explained by discontinuous *pillar* indices. When comparing anesthesiologists’ assessments of BIS or SE, the SE “errors” were clustered while BIS “errors” were more continuously distributed, maybe reflecting the non-continuous SE index value distribution. In another study, anesthesiologists tried to titrate anesthesia to an SE index corridor between 40 and 60, but had difficulties to not go below these limits or to get back into the limits from lower SE index values [37]. Ultimately, only 45% of the recorded values fell within the predetermined range. The skewed distribution leaning towards low index values is particularly evident in Supplemental Fig. 2 of the study, suggesting that achieving the desired depth of anesthesia as indicated by the Entropy module poses a challenge even when it is the primary target intervention of a prospective study. In a separate study, 76 patients in the intervention group received anesthesia based on the Surgical Pleth Index^TM^ (GE Healthcare) and SE, targeting an SE range of 40 to 60. Interestingly, the mean SE values for both the control and intervention groups leaned towards the lower end of the range. Specifically, during the period from after intubation to during surgery, the intervention group exhibited mean SE values ranging between 40–45, while the control group showed values between 39–43. No significant differences were observed between the groups [38]. While this evidence may be relatively modest, it is noteworthy that when aiming explicitly for a SE range of 40 to 60 on what is presumed to be a continuous scale, one might anticipate mean values closer to 50. Another group also noticed a strong tendency towards SE index values below 40 during anesthesia maintenance. The mean values reported for their measurement points correlate in parts with the *pillar* indices found in this study, i.e., a SE of 30, 37, or 41 [39]. These findings could suggest that the Entropy algorithm itself may generate skewed distributions, displaying a tendency toward lower index values. Schmidt and colleagues presented figures, where, similarly to our findings, index values with a higher probability of occurrence can be identified [40] and Vanluchene and colleagues described a smoother behavior for the BIS index values rather than SE [41]. However, even the BIS algorithm seems to generate peak values as well as sudden changes of index value occurrence at distinct boundary values [42].

### Key results

Researchers and clinical anesthesiologists should be aware that the reduction of the available EEG signal into an index leads to a strong loss of information. If, as is the case with the discussed commercial indices, the algorithms are not fully disclosed, the optimal assessment of a patient’s anesthetic level is hampered. Overall, these findings advocate for a broader perspective in monitoring practices, moving beyond an index-centered approach. The information provided by EEG-based monitoring devices can offer valuable insights for navigating anesthesia, yet the overall management should integrate all information available. Barnard and colleagues stated that the combination of using the index and being capable of interpreting the raw EEG may be the best strategy to safely navigate a patient under general anesthesia [43]. When relying on a single number, parameters that are better understandable – like the spectral edge frequency or the individually calculated spectral entropy – may be the preferential choice. EEG, in its own right, serves as a potent tool for evaluating the anesthesia state of patients, and in the future, shifting the focus away from proprietary indices in favor of assessing the raw EEG as well as more refined EEG-based monitoring tools, could be a more promising direction.

### Limitations

Due to its retrospective nature, this study has several limitations. We did not have access to raw EEG data or EEG band-power, which would have been valuable for a more in-depth investigation of *pillar* index values. Anesthesia regimes were not explicitly administered according to a predefined target range for SE and RE values (however, the manufacturer recommended range of 40 to 60 is included in the monitor output). This may introduce a potential confounding factor and could partially contribute to a higher probability of low index values. We further did not include an in-depth anaylsis of the relationship between SE/RE/BSR-index values and demographic parameters, drug concentrations, or intraoperative hemodynamic parameters. This decision was based on the fact that we are addressing a technical phenomenon within a universally applicable (*one-size-fits-all*) system, which, by default, does not consider these factors [4].

## Conclusion

Given the discontinuous behavior of SE and RE, it appears that there is not a consistent alignment of the anesthetic level on the index scale. To enhance comprehension of the processed indices, it is imperative that the algorithms employed are accessible to all. EEG is an essential information source for effective patient monitoring. Anesthesiologists should also possess the skills to interpret raw EEG data and its spectral representation to address any deficiencies in the processed indices.

## Supplementary Information

Below is the link to the electronic supplementary material.

Supplementary file 1 (pdf 1079 KB)
