# Supplementary material for: Continuity with caveats in anesthesia: state and response entropy of the EEG
Source: J Clin Monit Comput. 2024 Apr 3;38(5):1057–68. doi: 10.1007/s10877-024-01130-9 (PMC11427563; doi:10.1007/s10877-024-01130-9)
Supplement: Supplementary file 1 — (pdf 1079 KB) [file 10877_2024_1130_MOESM1_ESM.pdf]

Supplementary Information to *Continuity with Caveats in Anesthesia - State and Response Entropy of the EEG*

Journal of Clinical Monitoring and Computing.

Authors: Max Ebensperger, Matthias Kreuzer, Stephan Kratzer, Gerhard Schneider, Stefan Schwerin.

Corresponding author: Matthias Kreuzer, m.kreuzer@tum.de, Department of Anesthesiology and Intensive Care, School of Medicine and Health, Technical University of Munich, Ismaningerstr. 22, 81675 Munich, Germany

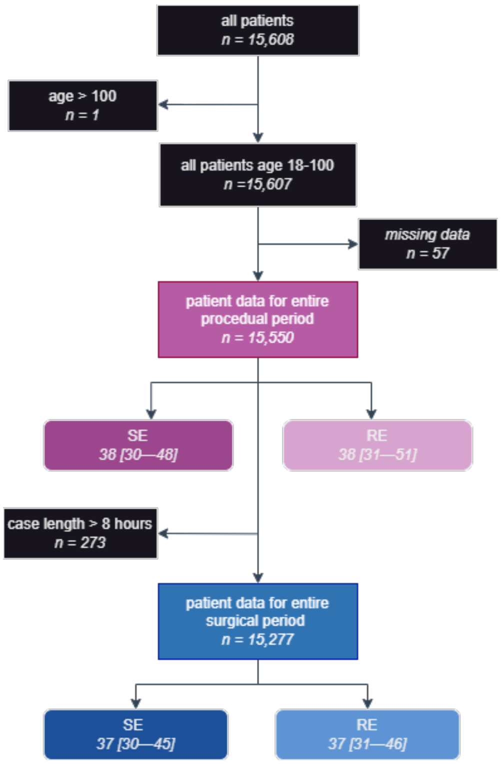

Fig. S1 Patient selection with State Entropy (SE) and Response Entropy (RE) index values

Flowchart with patient exclusion criteria as well as medians with Q1–Q3 of SE and RE index values for the entire procedural period and the surgical core period.

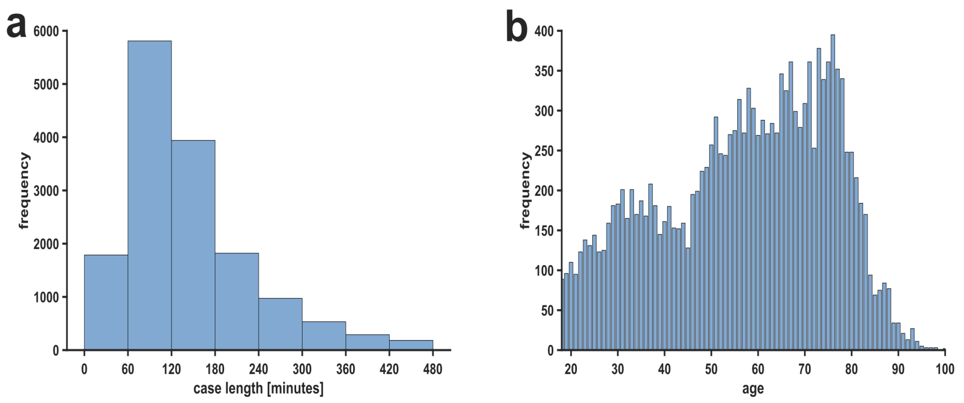

Fig. S2 Frequency distributions of surgery duration and patient age

a) Histogram of surgery time distribution of the included patients.

b) Histogram of the age distribution of the included patients.

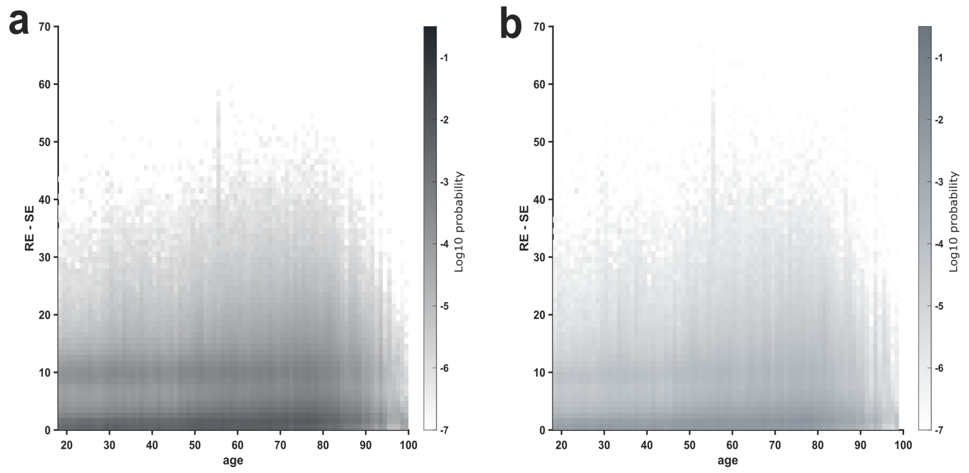

Fig. S3 Heat maps with the difference between Response Entropy (RE) and the State Entropy (SE)

By superimposing the heat maps, we can compute the disparity heat maps by subtracting the RE index values from the corresponding SE index values. This approach allows us to more effectively assess whether the data follows a continuous distribution pattern or if there are anomalies in occurrence frequency. From a visual perspective, it appears that the RE-SE values are distributed fairly evenly, with accumulations in the ranges of 0–5 and 9–12. Some discrepancies can be attributed to the overall scale of the values; as described before, SE index values only span from 0 to 91, whereas RE index values range from 0 to 100.

a) Heat map of RE-SE difference over the procedural period.

b) Heat map of RE-SE difference over the surgical period.

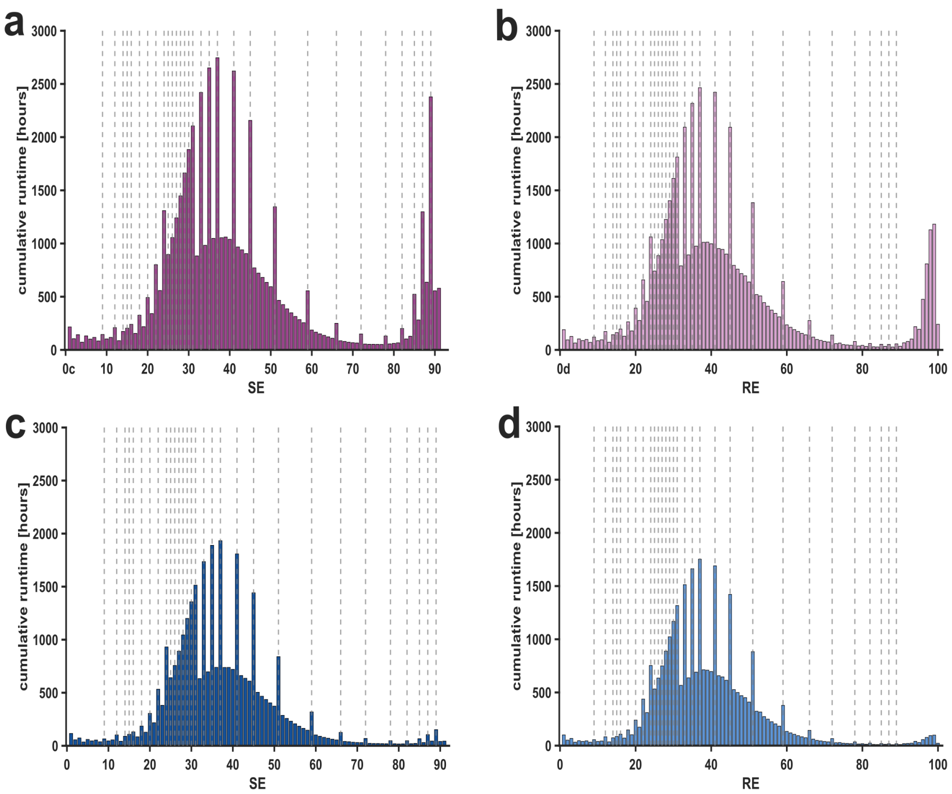

Fig. S4 Cumulative time spent in individual State Entropy (SE) and Response Entropy (RE) index values

The vertical dotted lines indicate the previously identified *pillar* index values. As expected, the highest cumulative time spent in certain index values overlaps with the described *pillar* index values.

a) Histogram of SE values over the procedural period

b) Histogram of RE values over the procedural period

c) Histogram of SE values over the surgical period

d) Histogram of RE values over the surgical period

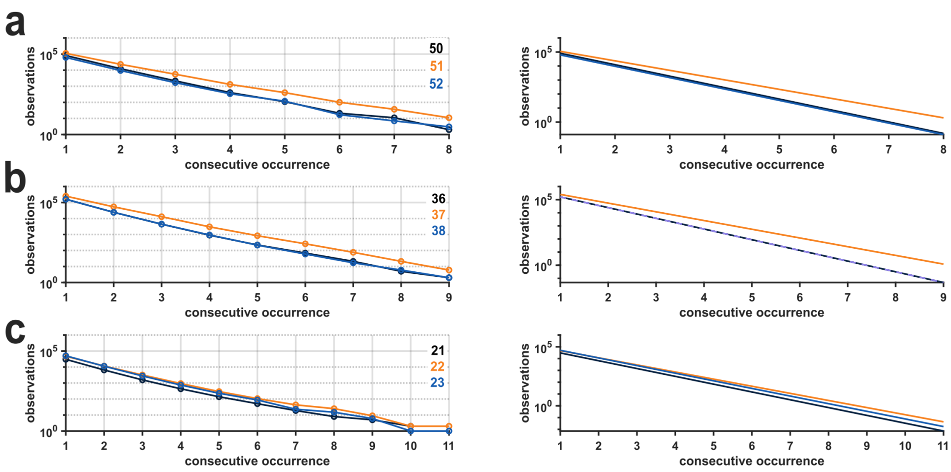

Fig. S5 Frequency of consecutive occurrences of exemplary State Entropy (SE) *pillar* index values in comparison to adjacent index values during surgical period

Three exemplary *pillar* SE index values are depicted in orange (51, 37, 22), with the adjacent index values in blue and black, which adhere to an expected continuous distribution. In terms of the frequency of consecutive occurrences, both the *pillar* index and adjacent index values are well-described by negative exponential functions. The corresponding fits were performed on the original data, not on log-transformed values. The y-axis was later logarithmically transformed for visual clarity. The plots on the right show these fits, revealing differences in both the rate of decay and the vertical shift.

a) *pillar* SE index value of 51 with adjacent index values on the left, with respective exponential fits on the right.

b) *pillar* SE index value of 37 with adjacent index values on the left, with respective exponential fits on the right.

c) *pillar* SE index value of 22 with adjacent index values on the left, with respective exponential fits on the right.

SE value	b	a	R2
50	535,582	-1.882	0.99998
**51**	**530,946**	**-1.560**	**0.99994**
52	402,029	-1.868	0.99998
36	1,048,716	-1.877	0.99997
**37**	**1,155,829**	**-1.529**	**0.99996**
38	1,048,638	-1.877	0.99997
21	138,686	-1.524	0.99995
**22**	**188,449**	**-1.389**	**0.99993**
23	222,848	-1.488	0.99996

Table S1 Parameters of the exponential fit as presented in **Figure** S5
The bold values in the table indicate the *pillar* indices. $y = e^{ax+b}$ where **b** determines the vertical shift of the function and **a** determines the steepness of the exponential decay. $\mathbf{R^2}$ is the variation explained by the fitted regression model, ranging from 0 to 1.0.

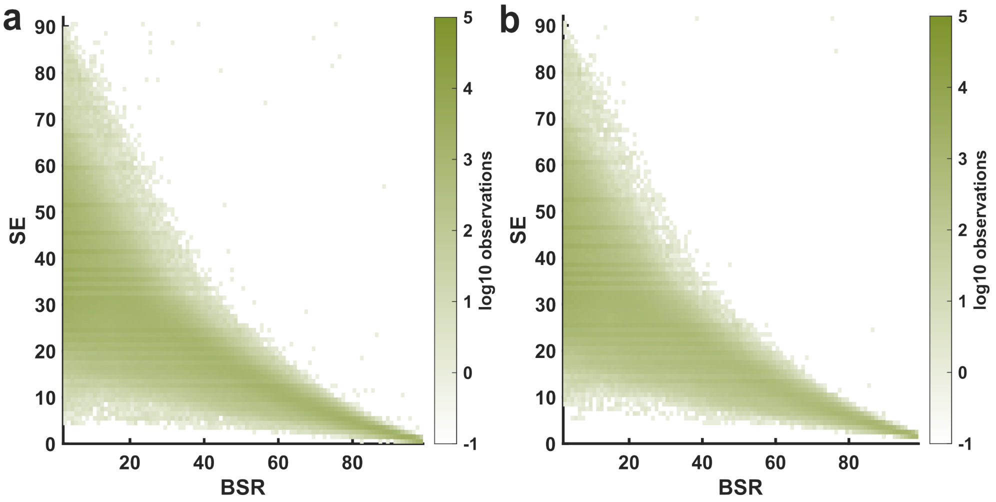

Fig. S6 Heat map showing the relationship between the Burst Suppression Ratio (BSR) and State Entropy (SE) throughout the procedural period and the surgical period
Prominent horizontal lines are evident, indicating the prevalence of *pillar* SE values. Conversely, the absence of vertical lines suggests that BSR *pillar* index values are not a significant feature in this data set.

a) Heat map for the procedural period.

b) Heat map for the surgical period.

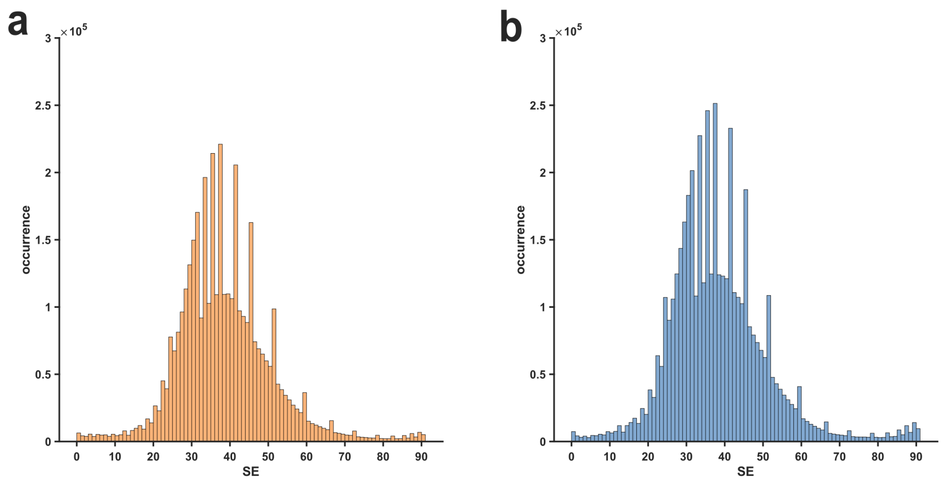

Fig. S7 Histogram showing State Entropy (SE) for volatile anesthetic gases and total intravenous anesthesia (TIVA)

The specific occurrence pattern observed in the *pillar* index values we presented for SE and RE remains unaffected by the administration of volatile anesthetic gases during anesthesia (a), as well as by the administration of anesthesia using intravenous drugs (b).

a) Histogram for volatile anesthetic gases

b) Histogram for TIVA
